# Cellular Fractionation and Nanoscopic X-Ray Fluorescence Imaging Analyses Reveal Changes of Zinc Distribution in Leaf Cells of Iron-Deficient Plants

**DOI:** 10.3389/fpls.2018.01112

**Published:** 2018-08-03

**Authors:** Gianpiero Vigani, Sylvain Bohic, Franco Faoro, Bart Vekemans, Lazlo Vincze, Roberto Terzano

**Affiliations:** ^1^Plant Physiology Unit, Department of Life Sciences and Systems Biology, University of Turin, Turin, Italy; ^2^Department of Agricultural and Environmental Sciences, Production, Landscape, Agroenergy, University of Milano, Milan, Italy; ^3^European Synchrotron Radiation Facility, NINA Beamline, Grenoble, France; ^4^Department of Analytical Chemistry, Ghent University, Ghent, Belgium; ^5^Department of Soil, Plant and Food Sciences, University of Bari, Bari, Italy

**Keywords:** cellular fractionation, iron, nanoscopic X-ray fluorescence imaging, zinc, plant

## Abstract

Multilevel interactions among nutrients occur in the soil-plant system. Among them, Fe and Zn homeostasis in plants are of great relevance because of their importance for plant and human nutrition. However, the mechanisms underlying the interplay between Fe and Zn in plants are still poorly understood. In order to elucidate how Zn interacts with Fe homeostasis, it is crucial to assess Zn distribution either in the plant tissues or within the cells. In this study, we investigated the subcellular Zn distribution in Fe-deficient leaf cells of cucumber plants by using two different approaches: cellular fractionation coupled with inductively coupled plasma mass spectrometry (ICP/MS) and nanoscopic synchrotron X-ray fluorescence imaging. Fe-deficient leaves showed a strong accumulation of Zn as well as a strong alteration of the organelles’ ultrastructure at the cellular level. The cellular fractionation-ICP/MS approach revealed that Zn accumulates in both chloroplasts and mitochondria of Fe deficient leaves. Nano-XRF imaging revealed Zn accumulation in chloroplast and mitochondrial compartments, with a higher concentration in chloroplasts. Such results show that (i) both approaches are suitable to investigate Zn distribution at the subcellular level and (ii) cellular Fe and Zn interactions take place mainly in the organelles, especially in the chloroplasts.

## Introduction

Among nutrients, iron (Fe) represents an essential element for the life cycle of plants, because it is a key cation to ensure electron flow in photosynthetic and respiratory pathways. For this reason, Fe can be a limiting factor for biomass production as well as for the quality of plant products. On the other hand, Fe is also potentially toxic because of its reactivity with oxygen, which catalyzes the formation of reactive oxygen species (Briat et al., 2015a).

The chlorosis observed in younger leaves represents the most typical visual symptom of Fe deficiency in plants. Indeed, Fe deficiency impairs chlorophyll synthesis, leading to interveinal chlorosis in developing leaves (Rodriguez-Celma et al., 2013) and decreased photosynthesis rates (Terry, 1980). However, the excess of several heavy metals can induce such chlorosis as well (Vert et al., 2002; Leskova et al., 2017), probably because Fe shares a number of similarities (i.e., electronic configuration, availability in the soil, or uptake mechanisms by plant) with other transition metals.

Accordingly, the changes in the Fe nutritional status of a plant are usually associated with changes in a given subset of metals, such as Mn, Zn, Cu, Mo, Co (Baxter et al., 2008a,b; Murgia and Vigani, 2015), pinpointing to the existence of a multi-level interaction among nutrients in plant. For example, a strong interaction between Fe and Mo has been recently characterized in cucumber plants (Vigani et al., 2017). Also, Tomasi et al. (2014) observed that zinc (Zn) accumulates in the leaves of Fe-deficient cucumber plants as compared to Fe-sufficient and Fe-resupplied plants.

Iron deficiency is often determined by the physico-chemical characteristics of the soil. Indeed, in alkaline and calcareous soils Fe is mainly present as insoluble oxi-hydroxide compounds leading to a very low bioavailability of Fe for plant uptake. To survive in these stress conditions, plants have developed adaptive mechanisms to cope with the low bioavailability of Fe in order to increase its acquisition (Tsai and Schmidt, 2017 and references therein). Dicots and non-graminaceous monocots have evolved a reduction-based mechanism known as Strategy I. The main responses induced by Fe deficiency in Strategy I species are the increase in (i) a Fe(III)-chelate reductase activity (FC-R), the function of which is the NAD(P)H-dependent reduction of the ferric to the ferrous form; (ii) the activity of a ferrous iron transporter (IRT1), and (iii) a H^+^-ATPase activity whose task is to extrude protons useful both to acidify the rhizosphere and to generate and maintain a transmembrane electrochemical gradient which facilitates the Fe^2+^ uptake (Tsai and Schmidt, 2017 and references therein).

IRT1 has a weak substrate specificity and contributes therefore to the accumulation of a broad range of divalent transition metals, including Zn (Vert et al., 2002; Arrivault et al., 2006; Haydon et al., 2012). Similar to Fe, Zn is still an essential microelement for cell life, being a crucial cofactor for all six classes of enzymes: oxidoreductases, hydrolases, transferases, lyases, isomerases, and ligases (Coleman, 1998). Moreover, Zn plays an important structural role in regulatory proteins (Berga and Shi, 1996). However, high concentrations of Zn can be toxic for the cell causing oxidative stress (Sresty and Madhava Rao, 1999).

Zn is a highly effective cofactor since its coordination geometry is highly flexible (Fraústo da Silva and Williams, 1991). For this reason, Zn is an important structural component of small protein motifs, named Zinc fingers, characterized by the coordination of one or more Zn ions in order to stabilize protein structures. Zinc finger motifs are known to bind several target ligands, for example, DNA.

Thanks to these properties, Zn can play different roles when interacting with proteins: (i) catalytic, where Zn ions directly participate in the reaction (e.g., carbonic anhydrase); (ii) co-catalytic, where Zn plays a catalytic role together with several metal ions that interact with each other in a co-catalytic Zn site (e.g., alkaline phosphatase); (iii) structural, where Zn ions stabilize the tertiary structure of the enzyme in a similar way as the disulphide bonds (e.g., DNA-binding proteins; alcohol dehydrogenase) (Vallee and Auld, 1990; Escudero-Almanza et al., 2012).

Despite plant symptoms under Zn excess resemble symptoms of Fe-deficient plants, the mechanism underlying the interplay between Fe and Zn in plants is poorly understood. Indeed, it is known that Zn excess causes physiological Fe deficiency, probably due to an alteration of Fe transport form soil. The ability to take up iron and zinc by IRT1 is inhibited by the addition of excess zinc and iron, respectively (Rogers et al., 2000).

In order to understand how Zn interacts with Fe homeostasis, it is crucial to know how Zn is distributed in the plant tissues and in the cells of Fe-deficient plants.

The distribution of metals in plants can be studied by various imaging techniques, using histochemical methods or analytical techniques employing electrons, charged particles, or X-ray beams (Terzano et al., 2013). Nevertheless, scanty information is still available concerning the Zn distribution within plant cells (Wu et al., 2005; Li et al., 2006). Some authors stated, indeed, that one hurdle to the study of subcellular zinc homeostasis is the lack of techniques applicable to the study of this spectroscopically silent ion (Blaby-Haas and Merchant, 2013). X-ray fluorescence (XRF) methods are powerful analytical techniques for non-destructive elemental analysis with the capability to detect trace level concentrations of multiple elements. At third-generation synchrotron facilities, submicrometer resolutions and parts-per-million (ppm) to parts-per-billion (ppb) minimum detection limits can be obtained within a feasible experimental time frame.

Micro X-ray florescence (μ-XRF) analyses on cucumber plants showed an amount of Zn more than double in leaf tissues of Fe deficient plants as compared to plants resupplied with natural Fe-sources (Tomasi et al., 2014). Subcellular elemental distributions in plant samples have been imaged by μ-XRF for As in *Ceratophyllum demersum* (Mishra et al., 2016); Cd and Zn in arbuscular mycorrhizas (Nayuki et al., 2014); and As, Fe, Zn, Mn, and macronutrients in *Oryza sativa* (Moore et al., 2014). However, in all these studies, the X-rays were focused to a beam size ranging from about 500 nm to 20 μm, thus not allowing a clear visualization of the cell’s internal structures and organelles. A new generation of synchrotron nanoprobes is nowadays available with ever-smaller spot sizes, down to a few tens of nanometers (Laforce et al., 2014). An example of such synchrotron X-ray nanoprobe is the newly developed ID16 NINA (nano imaging nano analysis) beamline at the European Synchrotron Radiation Facility (ESRF) of Grenoble (France) (Laforce et al., 2014).

In this work, we provide evidence about the localization and homeostasis of Zn at sub-cellular level in Fe-deficient cucumber leaves by mapping for the first time a plant cell with XRF at nanometers resolution, and by comparing XRF data with data from cell fractionation coupled to ICP/MS analysis.

## Materials and Methods

### Plant Material and Growth Conditions

Seeds of cucumber (*Cucumis sativus* L. cv. Marketer) were surface-sterilized and sown in Agriperlite, watered with 0.1 mm CaSO_4_, allowed to germinate in the dark at 26°C for 3 days, and then 70 seedlings were transferred to a box containing 20 L of the following nutrient solution: 2 mM Ca(NO)_3_, 0.75 mM K_2_SO_4_, 0.65 mM MgSO_4_, 0.5 mM KH_2_PO_4_, 10 μM H_3_BO_3_, 1 μM MnSO_4_, 0.5 μM CuSO_4_, 0.5 μM ZnSO_4_, 0.05 μM (NH_4_)Mo_7_O_24_; 0.1 mM Fe(III)-EDTA was added to control (+Fe) plants but omitted in Fe-deficient (–Fe) plants. The pH was adjusted to 6.0–6.2 with NaOH. Aerated hydroponic cultures were maintained in a growth chamber with a day:night regime of 16:8 h and a photosynthetic photon flux density (PPFD) of 200 μmol photons m^-2^ s^-1^. The temperature was 18°C in the dark and 24°C in the light. Expanded leaves of 10 days-old cucumber plants, grown in the presence (+Fe) and in the absence of Fe (-Fe) in the medium were harvested. As previously described, 10-days-old cucumber leaves of plants grown under Fe deficiency displayed evident chlorosis symptoms and the chlorophyll content decreased by about 80% with respect to +Fe leaves (Vigani et al., 2013a).

### Purification of Mitochondria and Chloroplasts

Mitochondria and chloroplasts were purified according to Rödiger et al. (2010) from expanded leaves of 10-days-old cucumber plants grown in the presence (+Fe) and in the absence of Fe (-Fe) in the medium. During purification steps, the following cellular fraction were harvested: total extract (TE, collected after homogenized tissues filtration), soluble fraction (SF, collected after mitochondrial precipitation and representing the cytosolic plus vacuolar lumen compartments), purified chloroplasts (Ch, collected at the interface between 35/80% percoll gradient), and purified mitochondria (Mit, collected at the interface between 23/40% percoll gradient) (**Supplementary Figure S1**). The purity degree was tested by Western blot analysis according to Vigani et al. (2015). Zinc and Fe content was determined on TE, SF, Ch, and Mit fractions.

### Ionomics

Sampled tissues were dried and then mineralized in HNO_3_ by using a Microwave Digestion System (Multiwave ECO). Chloroplast and mitochondrial fractions were mineralized in HNO_3_ at 100–120°C according to Vigani et al. (2017). Metal content was determined by inductively coupled plasma-mass spectrometry (ICP/MS, aurora M90 BRUKER) according to Vigani et al. (2013a, 2017). Data collected are from three independent experiments.

### Transmission Electron Microscopy

Samples of +Fe and -Fe expanded leaf tissues were fixed in a mixture of 3% (v/v) glutaraldehyde and 2% (w/v) paraformaldehyde in 0.1 mM phosphate buffer, pH 7, overnight at room temperature. Samples were subsequently post fixed with 1% (w/v) osmium tetroxide in the same buffer for 1 h at 4°C and dehydrated in a graded ethanol series before being embedded in SPURR resin (Electron Microscopy Sciences, Washington, PA, United States) according to Vigani et al. (2009). Ultrathin sections (100 nm thickness) were cut from at least three leaf samples from different plants and contrasted with uranyl acetate and lead citrate and examined with a Jeol JEM-100 SX TEM at 80 kV.

### Nano X-Ray Fluorescence Imaging

Ultrathin sections were prepared as described for TEM analyses with a few modifications: osmium post-fixation was omitted and thin sections (around 200 nm thickness) were stained with toluidine blue before mounting them on SiN support meshes for X-ray analysis (DuraSiN, distributed by Electron Microscopy Sciences). Staining was necessary to visualize the portion of the sample to analyze at the synchrotron facility through the use of an optical microscope.

The nano-XRF analyses were performed at the nano-imaging beamline ID 16A of the ESRF in Grenoble (France). The synchrotron X-rays were focused at an energy of 17 keV to a beam size varying from 100 to 20 nm (depending on the sample) by means of an X-ray focusing optics based on a Kirkpatrick–Baez mirror system with a multilayer surface coating. A six elements silicon drift diode detector was used to collect the fluorescence signal.

XRF data were analyzed by using PyMca software (version 5.1.1). Zinc distribution maps are displayed by using the same grayscale for all the samples so that they can be directly visually compared. The relative amount of Zn in chloroplasts and mitochondria was obtained from the XRF sum spectrum of each organelle by dividing the peak area of the Kα line of the element multiplied per 10^3^ for the area of the scatter signal, used to normalize the data for synchrotron current variations. The sum spectra were extrapolated from XRF maps by using PyMca and selecting the pixels forming the image of each organelle. All the spectra from each pixel were then summed to have the final sum spectrum. Eight chloroplasts and four mitochondria were considered for each sample. The number of mitochondria was lower because it was more difficult to distinguish them from other organelles (like peroxisomes) due to the low resolution of the XRF images, which did not allow to clearly distinguish the internal structures for all the organelles.

## Results

### Transmission Electron Microscopy

**FIGURE 1:**
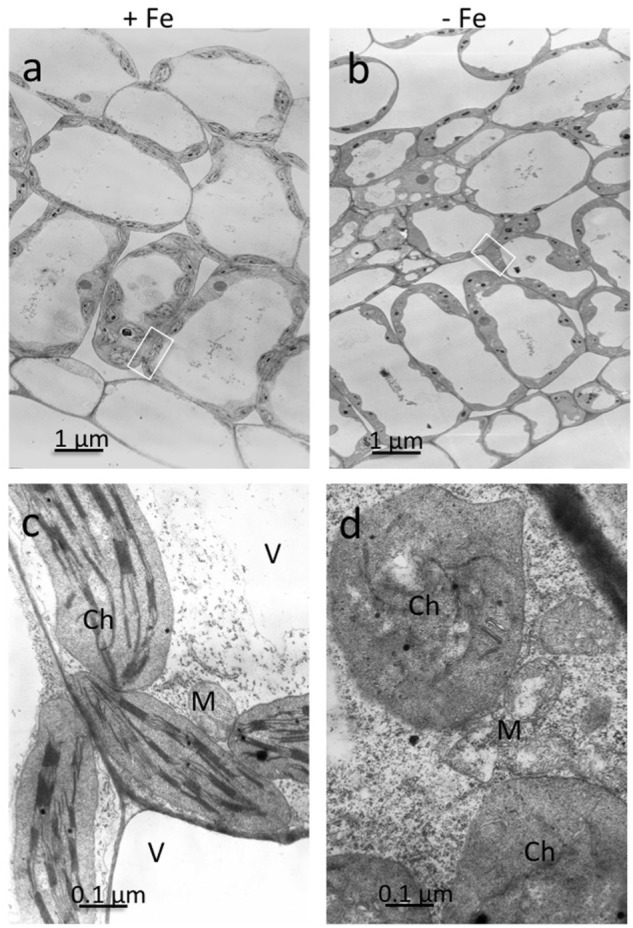
Transmission electron microscopy (TEM) analysis of leaf tissues from cucumber plants grown in control condition (+Fe) or under Fe deficiency (–Fe). Control leaf tissues **(a,c)** and –Fe leaf tissues **(b,d)**. All scale bars = 1 μm **(a,b)** 0.1 μm **(c,d)**. M, mitochondrion; Ch, chloroplast; V, vacuole.

Ultrathin sections obtained from mesophyll of +Fe and –Fe cucumber leaves were observed by TEM at different magnifications (**Figure 1**). From these observations, it is evident that the most striking morphological alteration in -Fe leaf tissues is related to chloroplast ultrastructure (**Figures 1b,d**). The organelles appeared swollen and some of them almost roundish with a dense stroma and a very low number of thylakoids, usually not organized in grana (**Figure 1d**). In some instance, also mitochondria showed altered organization with reduced number of cristae (**Figure 1d**), while no other apparent cytopathic effect was detectable in cell ultrastructure, in comparisons with +Fe tissue (**Figures 1a,c**).

### Leaf Ionome Profile

To evaluate the metabolic consequences of induced Fe deficiency, ionome of +Fe and -Fe plants was profiled by quantifying the content of the macronutrients sodium (Na), magnesium (Mg), potassium (K), calcium (Ca) as well as of the micronutrients manganese (Mn), zinc (Zn), copper (Cu), Fe and Mo in leaves (**Table 1**).

**Table 1:** Na, Mg, K, Ca, Mn, Fe, Cu, Zn, and Mo concentration (μg/gDW) in leaves of cucumber plants grown under Fe-sufficient (+Fe) and Fe-deficient (-Fe) conditions.

	+Fe	-Fe	-Fe/+Fe (%)
Na	84.81 @ 31.94	122.96 @ 32.86	–
Mg	5478.54 @ 1176.69	11,988.53 @ 3150.08*	+119
K	34,312.26 @ 126.55	61,947.68 @ 7.59*	+81
Ca	19,925.31 @ 11,504.09	17,731.98 @ 6616.97	–
Mn	30.31 @ 8.72	75.23 @ 27.65*	+148
Fe	166.49 @ 30.88	68.95 @ 24.96*	**-59**
Cu	11.97 @ 2.05	29.01 @ 4.42*	+142
Zn	31.75 @ 8.44	113.64 @ 16.40*	**+258**
Mo	15.26 @ 0.91	7.26 @ 0.21*	-52

*Value significantly different (*p* < 0.05) in Student’s *t*-test are indicated with^∗^. Percentage changes of Fe and Zn content between +Fe and -Fe treatment are highlighted in bold.*

As expected, the growth of –Fe plants modulated Fe concentration, as seen by the reduced Fe content (by about 59%) in -Fe leaves when compared with +Fe ones.

The increased levels of K, Mg, Mn, Zn, and Cu, under Fe deficiency, indicate that these elements are dependent on Fe availability in leaves, while Na and Ca are not affected (**Table 1**). Moreover, Mo levels decreased in -Fe leaves relative to control leaves. The ionome profile of the leaves revealed that under Fe-deficiency Zn was the element which displayed the highest overall increase respect to the +Fe plants.

### Cellular Fractionation-ICP/MS Analyses

**FIGURE 2:**
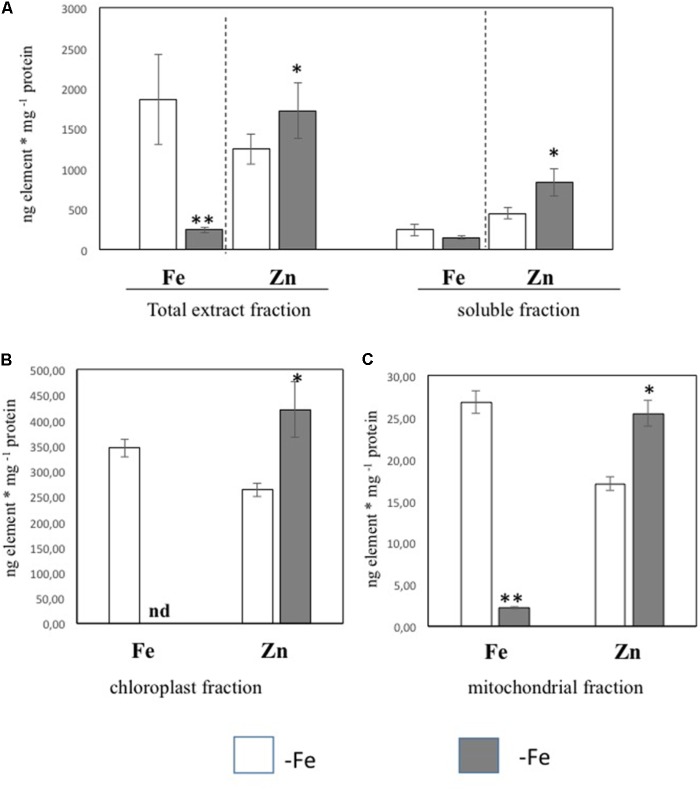
Fe and Zn concentration in total protein extract and soluble protein fractions **(A)** and in chloroplast **(B)** and mitochondrial **(C)** fractions purified from leaf tissues of cucumber plants grown in control condition (+Fe) or under Fe deficiency (–Fe). Values, expressed as ng element ^∗^ mg –1 protein are the means ± SE of three independent samples. Value significantly different *p* < 0.05 or *p* < 0.01 in Student’s *t*-test are indicated with ^∗^ and ^∗∗^, respectively.

In order to further investigate the Zn distribution in -Fe cells in relation to Fe, the subcellular distribution of Fe and Zn was assessed by ICP/MS analysis on four different cellular fractions collected from leaf tissues of +Fe and –Fe plants: TE, SF, Ch, and Mit (see Materials and Methods section and **Supplementary Figure S1**). Significant differences in protein yield for each fraction between +Fe and –Fe samples were not observed, as the variability among biological replicates was high. Forty grams of fresh weight of leaf material (regardless of treatments) yielded proteins amount of 0.9–1.8 g TE, 0.8–1.5 g SF, 1–3 mg Ch, and 70–100 μg Mit [i.e., 23–45 mg TE protein per gram of fresh weight (mg/g), 20–38 mg/g of SF proteins, 250–300 μg/g of Ch proteins, and 1.7–2.5 μg/g of Mit proteins]. In the TE fraction, a significant decrease of Fe concentration (expressed as ng/mg of protein) as well as a significant increase of Zn was detected in –Fe samples, while in the SF only a significant increase of Zn concentration was observed (**Figure 2A**). Interestingly, Fe concentration did not change in –Fe respect to +Fe in SF samples. Under Fe deficiency, Zn accumulated in both chloroplast and mitochondrial-enriched fractions (**Figures 2B,C**). However, in –Fe samples, Fe content in the chloroplasts and mitochondria dramatically decreased as compared to +Fe plants, being below the detection limit in chloroplasts, and reduced by 90% in the mitochondria (**Figures 2B,C**). Although possible contamination among fractions might occur under cellular fractionation, such analysis revealed that under Fe deficiency: (i) Fe content was greater (at least detectable) in mitochondria than in chloroplasts; and (ii) Zn accumulated more in chloroplasts than in mitochondria.

### Nano XRF Imaging of Leaf Cells and Zn Distribution in Chloroplasts and Mitochondria

**FIGURE 3:**
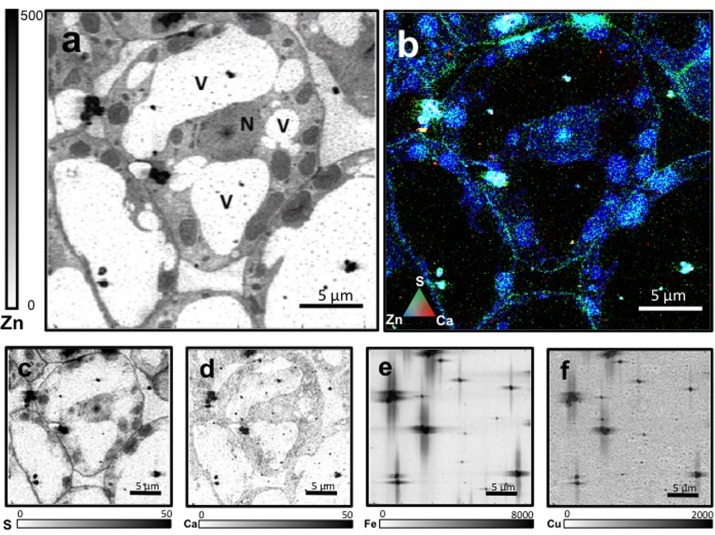
Nano X-ray fluorescence (K_α_ lines) metal distribution maps of Fe-deficient leaf cells for Zn **(a)**, S **(c)**, Ca **(d)**, Fe **(e)**, and Cu **(f)**. Darker pixels correspond to higher elemental concentrations. Scale bars based on counts are reported for each element. An RGB image of the distribution of Zn (blue), S (green), and Ca (red) is also presented **(b)**. N, nucleus; V, vacuole.

**FIGURE 4:**
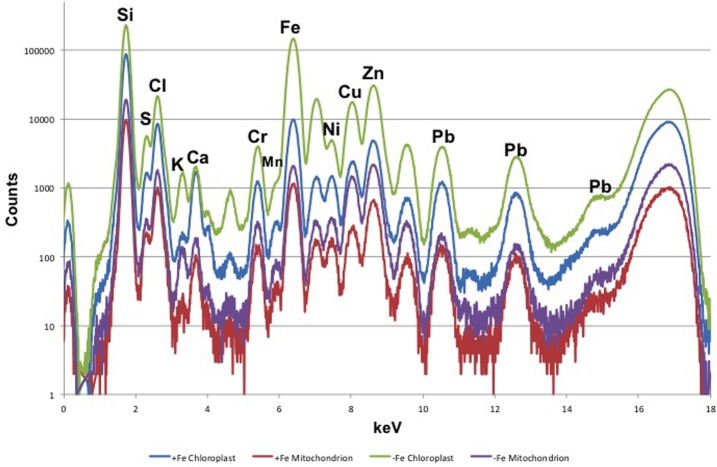
XRF sum spectra of single chloroplasts and mitochondria from Fe-deficient (–Fe) and Fe-sufficient (+Fe) plants.

**FIGURE 5:**
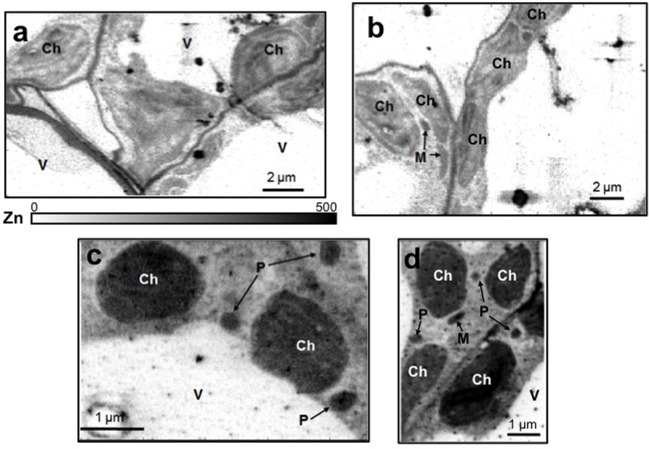
Subcellular Zn distribution maps (XRF K_α_ lines) within leaf cells for +Fe **(a,b)** and –Fe **(c,d)** samples. Darker pixels correspond to higher elemental concentrations. A scale bar based on counts is also reported. M, mitochondrion; Ch, chloroplast; P, peroxisome; V, vacuole.

Leaf mesophyll cells were imaged by nano-XRF and elemental distribution maps were obtained as reported in **Figure 3** for a Fe-deficient plant. Clear distribution maps were obtained only for few elements: Zn (**Figure 3a**), S (**Figure 3c**), and Ca (**Figure 3d**). An RGB map showing all the three elements together is also reported in **Figure 3b**. For other elements like Fe (**Figure 3e**) and Cu (**Figure 3f**), no subcellular distribution was visible except for contaminating particles (also visible in the Zn, S, and Ca maps), likely deriving from the staining process or atmospheric particulate contamination. Despite this contamination, while for certain elements like Fe and Cu, the concentration within the cell was too low to allow distribution mapping by this technique, Zn distribution appears to be not affected by staining and/or particulate except for the presence of highly concentrated spots, corresponding to the same hotspots of Fe, Cu, S, and Ca (**Figure 3**). Sum spectra of single chloroplasts and mitochondria from -Fe and +Fe plants are presented in **Figure 4**. Beside Zn, S, Ca, Fe, and Cu, other elements were detected: Si, Cl, K, Mn, Ni, and Pb. The Si signal derives from the SiN mesh used as support for the ultrathin sections. Pb signal is due to the staining solution used to allow the visualization of the cell structures within the thin section. Sum spectra showing the elemental composition of cytoplasm as compared to the full investigated areas (also containing organelles and vacuoles) are presented as **Supplementary Figure S2**. **Figure 5** shows Zn distribution maps within leaf organelles for +Fe (**Figures 5a,b**) and -Fe (**Figures 5c,d**) samples. As can be seen, Zn concentration in all the organelles of -Fe plants is higher than that in +Fe plants (darker pixels correspond to higher concentrations). The distribution maps of S, Ca, Fe, and Cu in the same areas are presented as **Supplementary Figure S3**. Chloroplasts are clearly discernible because of their large size and typical internal structures (thylakoid membranes are clearly visible). Peroxisomes appear as small rounded objects while mitochondria show a more elongated shape (**Figure 5**).

**FIGURE 6:**
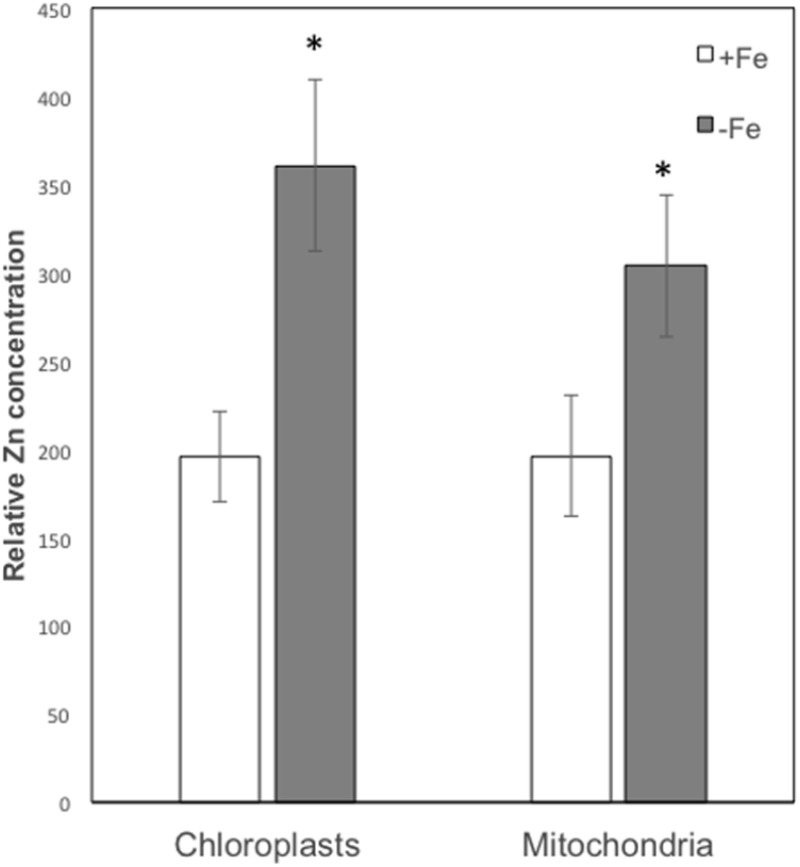
Zn relative concentration (±SD) in chloroplasts and mitochondria of +Fe and –Fe samples, as determined from the XRF sum spectra of each organelle. Value significantly different (*p* < 0.05) in Student’s *t*-test are indicated with ^∗^.

On the basis of their characteristic size and shape, various chloroplasts and mitochondria were identified in different cells and the XRF sum spectra of each organelle were used to extrapolate Zn relative concentration. As can be seen from **Figure 6**, both in chloroplasts and mitochondria Zn concentration is higher in -Fe plants compared to +Fe plants. In particular, Zn concentration in -Fe chloroplasts was about 85% higher than in control +Fe chloroplasts and about 55% higher in mitochondria. These data thus confirm what was observed with cell fractionation followed by ICP/MS analyses, i.e., a higher increase of Zn concentration in chloroplasts compared to mitochondria in -Fe plants.

## Discussion

In this work, we aimed to provide new evidences about the interplay between Fe and Zn in plants by characterizing the subcellular localization of accumulated Zn in leaf tissues of Fe-deficient cucumber plants. The investigation of subcellular Zn homeostasis in plants is quite difficult as specific techniques applicable for such ion are still lacking (Blaby-Haas and Merchant, 2013).

Therefore, we compared the results from two different analytical approaches in order to assess Zn distribution at sub-cellular level under Fe deficiency: ion determination by ICP/MS technique on different cellular fractions separated by ultracentrifugation, and nanoscopic XRF imaging method.

ICP/MS has been established as the most reliable technique for quantifying metals, metalloids, and some non-metals in a wide range of samples with a wide working range of concentrations, high sensitivity, and low interferences. The use of ICP/MS, by allowing simultaneous measurement of multiple elements in the same sample tissue, has significant advantages for biological applications. However, few reports analyzed the metal content at subcellular level in plants (Tan et al., 2010; Vigani et al., 2017).

According to the data obtained by cellular fractionation and organelles purification followed by ICP/MS analysis, under Fe deficiency the Fe content decreased in TE, Ch, and Mit cellular fractions, while Zn content increased in all the cellular fractions collected. In particular, Fe content in chloroplasts and mitochondria was strongly reduced, with a decrease of 90% in mitochondria and close to 100% in chloroplasts, not being detected anymore by ICP/MS. It is well known that chloroplasts represent the major sink of Fe in leaf cells (Nouet et al., 2011; Vigani et al., 2013b). It has been estimated that 68% of the Fe present in the vegetative shoot of Arabidopsis plant was found to be in the chloroplast (Shikanai et al., 2003). Therefore, our results suggest that Fe deficiency weakens Fe content more in chloroplasts than in mitochondria or, alternatively, that under Fe starvation conditions mitochondria might have a priority for Fe allocation with respect to chloroplasts. Accordingly, in the green microalgae, mitochondria seem to be more resistant than chloroplast to Fe deficiency, suggesting a preference for Fe delivery to mitochondria when Fe is low (Nouet et al., 2011). Additionally, it has been stated that chloroplast play a central role in the regulation of Fe economy strategy of the cell under Fe deficiency in *Chlomydomonas reinhardtii* (Page et al., 2012). Recently, Hantzis et al. (2018) observed that in Arabidopsis Fe deficiency first affect photosynthesis than respiration, suggesting that photosynthesis is more dispensable under Fe deficiency compared to respiration. Accordingly, our results along with those provided by Hantzis et al. (2018) demonstrated that chloroplast is the main target of Fe deficiency highlighting the hypothesis that respiration might be preserved compared to photosynthesis under such nutritional stress.

Additionally, ICP/MS analysis revealed that Zn accumulation under Fe deficiency was significant both in chloroplast and in mitochondrial compartments. Therefore, the opposite behavior between Fe and Zn was observed in both organelles: when the Fe content decreased, the content of Zn increased. Interestingly, this did not occur in the SF, suggesting that both organelles may represent important cellular compartments where Fe and Zn interplay take place.

Nanoscopic XRF Imaging analyses of cucumber leaf confirmed that Zn accumulate mainly in chloroplasts and mitochondria of -Fe plants. Unfortunately, Fe distribution was not detectable because of the low concentration and the lower sensibility of the technique for this element, as compared to Zn. Beside the higher fluorescence yield of Zn (0.435) compared to Fe (0.32), also the energy of the X-rays used in the experiment (17 keV) favored the excitation of Zn atoms (9.657 keV) rather than Fe ones (7.109 keV). Overall, our results showed that Fe deficiency significantly affects the distribution of Zn in leaf cellular compartments: in particular chloroplast might represent a Zn sink under Fe deficiency.

An important issue to consider when dealing with synchrotron XRF analyses is the possible creation of artifacts. In this type of experiments, artifacts may derive mainly from sample preparation and photon damage (Lombi et al., 2011; Zhao et al., 2014; Kopittke et al., 2017). To image subcellular structures, ultrathin sectioning is needed as well as high flux synchrotron X-ray sources (to focus a sufficient number of photons on nm-sized spots). Therefore, we cannot completely exclude that artifacts were created during sample preparation and/or analysis. However, differently from Fe and Cu, it seems that Zn distribution was not biased by these issues, as it can be argued from the clean distribution maps and the relative Zn concentrations in -Fe and +Fe samples, which confirm the ICP/MS data.

The interplay between Fe and Zn has been already observed in different plants, but the specific molecular mechanism underlining such interaction is less investigated (Leskova et al., 2017). Indeed, it is known that at the root level Zn competes with Fe for the uptake via IRT1 (Korshunova et al., 1999). Additionally, it has been observed that foliar Fe supply strongly suppressed the development of chlorosis upon excess Zn and reduced shoot Zn accumulation (Leskova et al., 2017). Such results were not observed when foliar Fe was supplied to *irt1* mutant grown under Zn excess, suggesting a probably shoot-derived interaction between Fe and Zn, which might be relevant for the induction of Fe deficiency symptoms instead of the IRT1-mediated Zn uptake. Recently, it has been stated that the activation of Fe deficiency responses of plants growing under excess Zn were caused by an antagonist interaction between Zn and Fe (Leskova et al., 2017). In plants, Zn-dependent processes are located in all cellular compartments, including mitochondria (Heazlewood et al., 2004), and chloroplasts (Blaby-Haas and Merchant, 2013). To date, the requirement for Zn inside plant mitochondria has been linked to its role in important processes such as the degradation of mitochondrial presequences (Moberg et al., 2003; Tan et al., 2010). In this contest, several Zn-dependent metalloproteases have been identified in plant mitochondria (Moberg et al., 2003). Additionally, Zn is involved in the mitochondrial RNA editing and in in the small TIM (Translocase of the Inner Membrane) folding, and it is also requests by the COX4 subunit of cytochrome *c* oxidase in plant mitochondria (Vigani and Hanikenne, 2018 and references therein).

The role of Zn in chloroplasts has been overlooked because of its inability to perform redox chemistry. However, the interest in understanding Zn homeostasis in plant is gaining prominence (Sinclair and Kramer, 2012). Blaby-Haas and Merchant (2013) summarized the metal-dependent metabolic pathways in chloroplasts by considering different publicly available data. Chloroplast Zn-requiring enzymes are Superoxide dismutase (Cu/Zn), Alkenal/one oxidoreductase, Carboxy-transferase beta subunit of the acetyl-CoA carboxylase, proteases (FtsH proteins, peptidase M50 family protein, stromal processing peptidase), Zinc-finger proteins (involved in chloroplast and palisade cell development, in the ribosomal subunits, in the cyclic electron flow and chloroplast splicing factor). Therefore, several Zn-requiring proteins are localized in chloroplasts. Considering that Fe deficiency negatively impacts on morphology and functions of both chloroplasts and mitochondria, it is plausible to think that an increased requirement of Zn might sustain ROS detoxification and proteins degradation processes. However, further studies are needed in order to understand the specific role of Zn under Fe deficiency in such cellular compartments.

## Conclusion

Understanding nutrient interactions is a challenging and relevant issue in the plant nutrition field as it might reveal useful insights to develop new plant genotypes with improved mineral use efficiency and for biofortification programs (Briat et al., 2015b).

Iron and Zn represent important targets for such programs and several evidences reveal a strong interaction among these nutrients in plants. An impaired balance between Fe and Zn is perceived as Fe deficiency by plant. In order to identify the cellular site where such interaction take place we determined the subcellular distribution of Zn in -Fe leaf cells and we observed that Zn strongly accumulates in the cells of the leaves of -Fe cucumber plants. By comparing cellular fractionation-ICP/MS and nanoscopic XRF imaging results, we showed that under Fe-deficiency Zn accumulates both in chloroplasts and in mitochondria, with a higher level in chloroplasts. Our findings highlight the importance of focusing future investigations on Fe and Zn interaction at the subcellular level, especially on chloroplasts and mitochondria.

However, in order to reduce the possibility of artifacts creation during sample preparation and/or analysis, two possible approaches should be considered in future nanoscopic XRF experiments: (i) frozen-hydrated samples can be prepared by a fast-freezing process, cut as thin sections by using a cryomicrotome, and kept at low temperature until the analysis. Cryopreparations are usually less disturbing and limit the redistribution of metals and changes in speciation. However, also beamlines equipped with cryostages or cryochambers are then needed to preserve the frozen-hydrated state during the analysis. (ii) In order to avoid chemical staining, fast preliminary maps of larger areas in the sample can be acquired to locate the areas of interest prior to longer scans of the subcellular structures. For this purpose multi-crystal detector arrays, like the Maia detector (Kirkham et al., 2010), could dramatically reduce the sample exposition time thus reducing the risks of beam damage.

## Author Contributions

GV and RT conceived and designed the study. GV performed the cellular fraction analysis. FF carried out TEM analysis and revised the manuscript. SB, BV, LV, GV, and RT carried out nano XRF imaging analysis. GV and RT drafted the article. FF, SB, BV, and LV revised it critically. All the authors approved the final version of the article.

## Conflict of Interest Statement

The authors declare that the research was conducted in the absence of any commercial or financial relationships that could be construed as a potential conflict of interest.

## Supplementary Material

FIGURE S1 Workflow of the isolation of chloroplast and mitochondria from leaf tissues. Tube after percoll gradient for chloroplast (left) and mitochondria (right) purification are reported. Cellular fractions collected and analyzed are highlighted in yellow.

FIGURE S2 XRF sum spectra of the whole area imaged in **Figure 5b** (+Fe Full) and 5C (-Fe Full) in comparison with sum spectra of the cytosol extracted from pixel analysis of **Figure 5b** (+Fe Cytoplasm) and **Figure 5c** (-Fe Cytoplasm).

FIGURE S3 Subcellular distribution maps (XRF K_α_ lines) of S, Ca, Fe, Cu within leaf cells for +Fe **(A,B)** and -Fe **(C,D)** samples, as in **Figure 5** of the main text. Darker pixels correspond to higher elemental concentrations. A scale bar based on counts is also reported.
