# Supplementary figures and images for: Cellular Fractionation and Nanoscopic X-Ray Fluorescence Imaging Analyses Reveal Changes of Zinc Distribution in Leaf Cells of Iron-Deficient Plants
Source: Front Plant Sci. 2018 Aug 3;9:1112. doi: 10.3389/fpls.2018.01112 (PMC6085429; doi:10.3389/fpls.2018.01112)

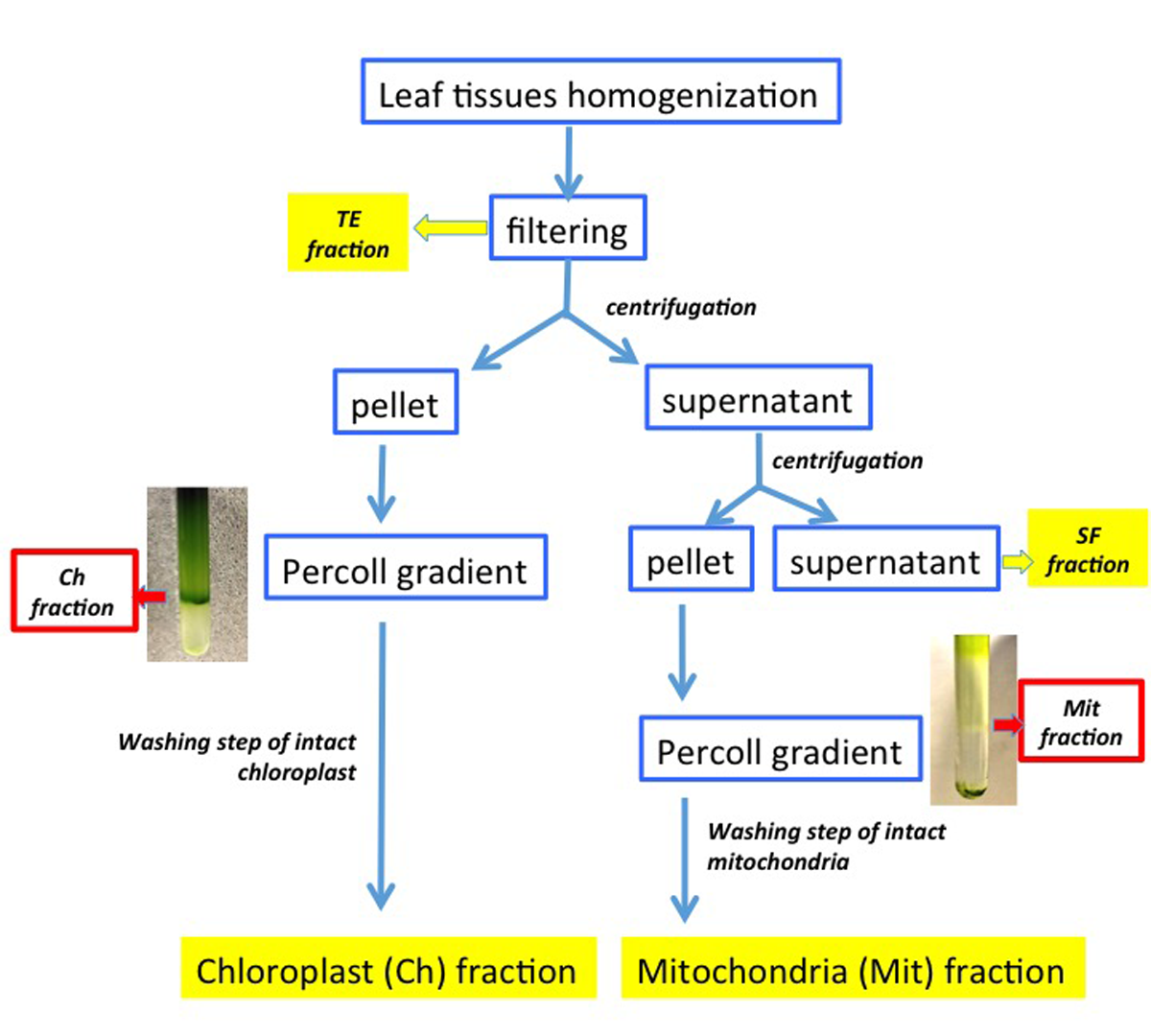

Supplement: FIGURE S1 — Workflow of the isolation of chloroplast and mitochondria from leaf tissues. Tube after percoll gradient for chloroplast (left) and mitochondria (right) purification are reported. Cellular fractions collected and analyzed are highlighted in yellow. [file Image_1.TIF]

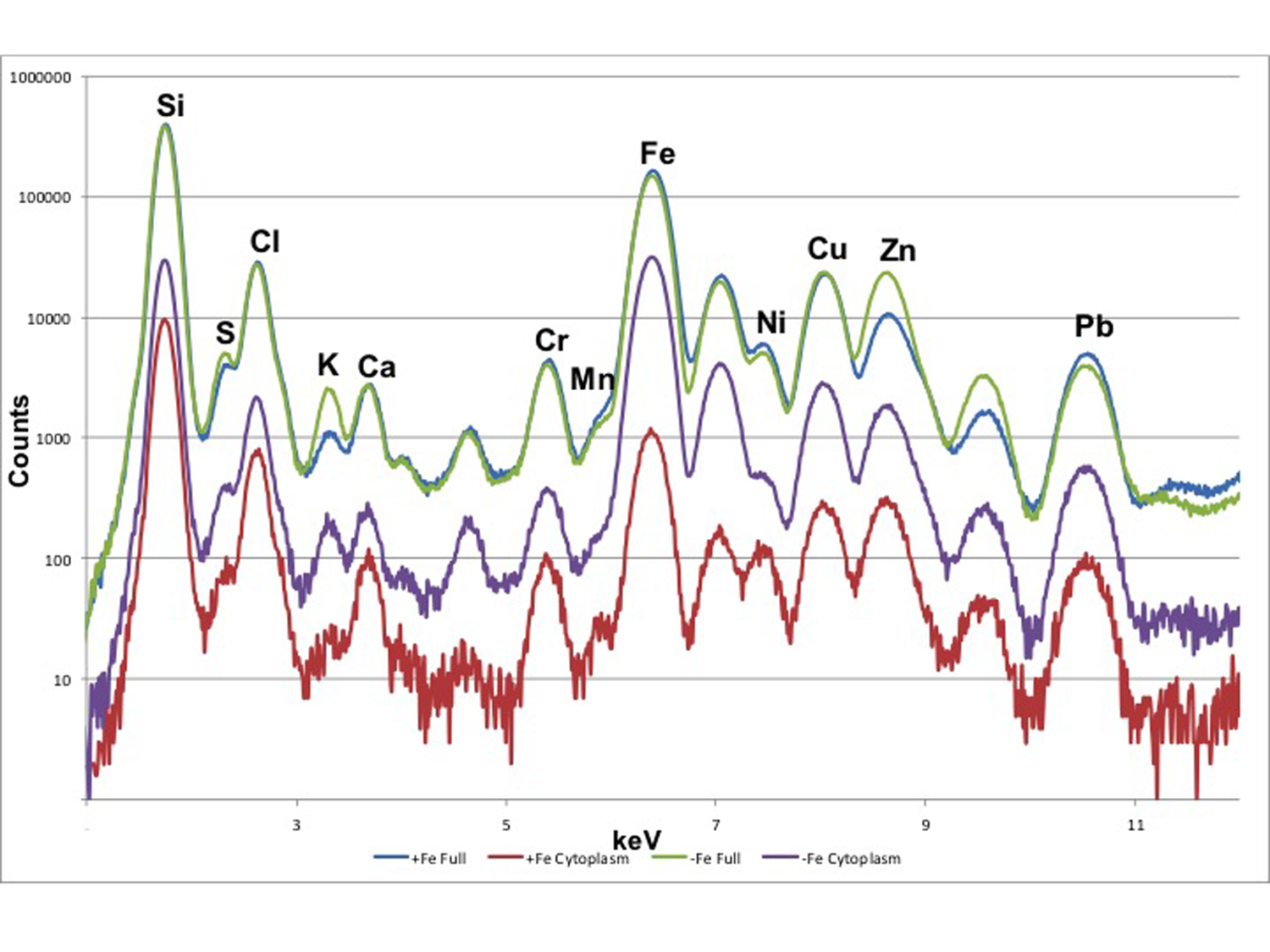

Supplement: FIGURE S2 — XRF sum spectra of the whole area imaged in Figure 5b (+Fe Full) and 5C (-Fe Full) in comparison with sum spectra of the cytosol extracted from pixel analysis of Figure 5b (+Fe Cytoplasm) and Figure 5c (-Fe Cytoplasm). [file Image_2.TIF]

**a**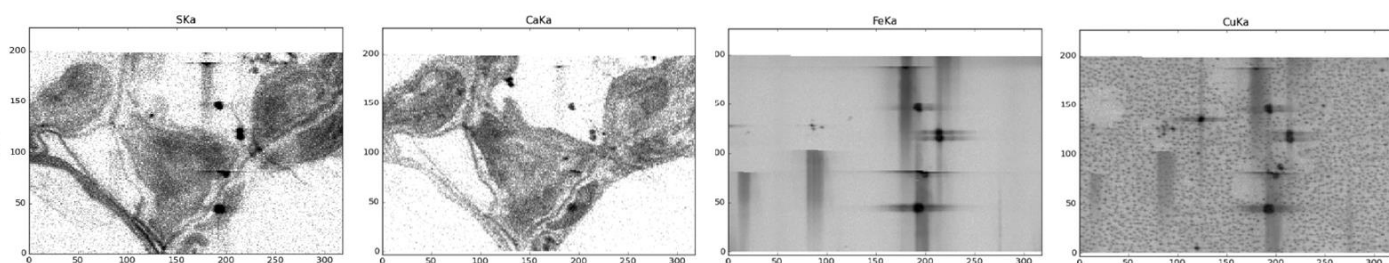**b**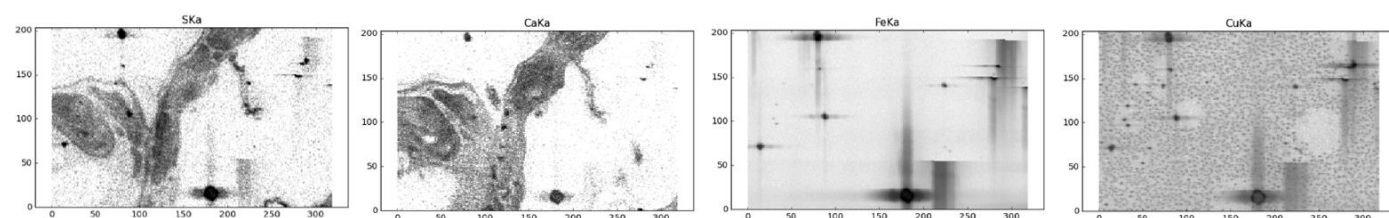**c**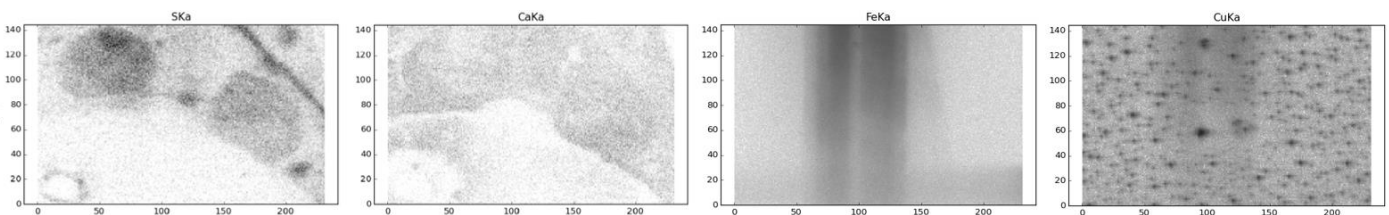**d**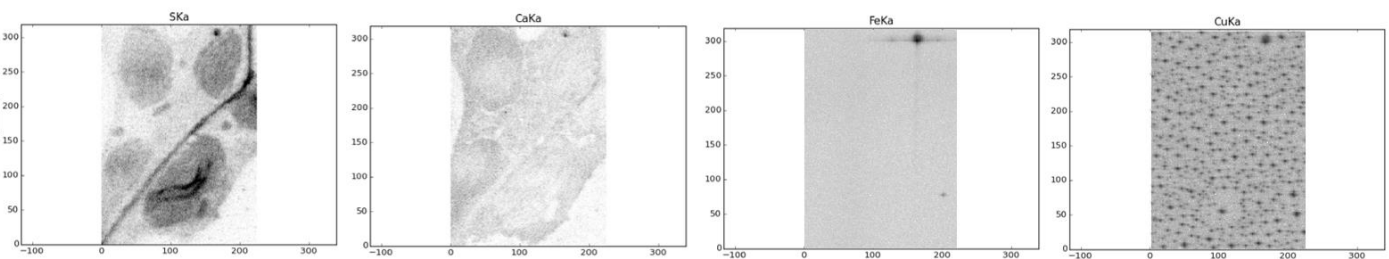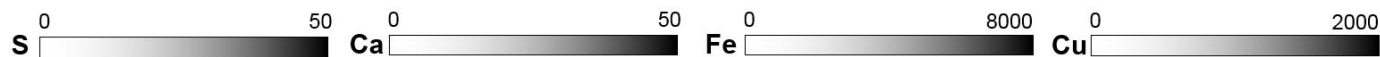

Supplement: FIGURE S3 — Subcellular distribution maps (XRF Kα lines) of S, Ca, Fe, Cu within leaf cells for +Fe (A,B) and -Fe (C,D) samples, as in Figure 5 of the main text. Darker pixels correspond to higher elemental concentrations. A scale bar based on counts is also reported. [file Image_3.pdf]
